# Proteomic Alterations in B Lymphocytes of Sensitized Mice in a Model of Chemical-Induced Asthma

**DOI:** 10.1371/journal.pone.0138791

**Published:** 2015-09-23

**Authors:** Steven Haenen, Jeroen A. J. Vanoirbeek, Vanessa De Vooght, Liliane Schoofs, Benoit Nemery, Elke Clynen, Peter H. M. Hoet

**Affiliations:** 1 Centre for Environment and Health, KU Leuven, Leuven, Belgium; 2 Research Group Functional Genomics and Proteomics, KU Leuven, Leuven, Belgium; 3 Biomedical Research Institute (BIOMED), Hasselt University, Hasselt, Belgium; Leiden University Medical Center, NETHERLANDS

## Abstract

**Introduction and Aim:**

The role of B-lymphocytes in chemical-induced asthma is largely unknown. Recent work demonstrated that transferring B lymphocytes from toluene diisocyanate (TDI)-sensitized mice into naïve mice, B cell KO mice and SCID mice, triggered an asthma-like response in these mice after a subsequent TDI-challenge. We applied two-dimensional difference gel electrophoresis (2D-DIGE) to describe the “sensitized signature” of B lymphocytes comparing TDI-sensitized mice with control mice.

**Results:**

Sixteen proteins were identified that were significantly up- or down-regulated in B lymphocytes of sensitized mice. Particularly differences in the expression of cyclophilin A, cofilin 1 and zinc finger containing CCHC domain protein 11 could be correlated to the function of B lymphocytes as initiators of T lymphocyte independent asthma-like responses.

**Conclusion:**

This study revealed important alterations in the proteome of sensitized B cells in a mouse model of chemical-induced asthma, which will have an important impact on the B cell function.

## Introduction

In classic allergic asthma, an important role is attributed to specific IgE antibodies [1,2]. Specific IgE is produced after isotype class switching of B cells to plasma cells. According to the most common paradigm, this response is initiated by antigen presentation to T cells, followed by T cell activation and secretion of cytokines to activate B lymphocytes. Recently, evidence has grown that antibody secretion may only be one facet of B cell function. This was first demonstrated by Drolet *et al*. who showed T cell independent B cell functions [3]. Lindell *et al*. showed that pulmonary B cells from mice, chronically challenged with cockroach antigen (CRA), can present the antigen to CD4^+^ T cells resulting in Th2 cytokine production *in vitro* [4]. In lungs and bronchoalveolar lavage (BAL) fluid of CRA-challenged mice without B cells (B cell KO), significantly lower levels of Th2 cytokines, IL-4 and IL-5, were detected compared to wildtype mice (control) and this was accompanied by an attenuation of airway hyperresponsiveness (AHR), while inflammatory cells remained present in the lungs of B cell KO mice following chronic CRA challenge compared to control mice.

It is suggested that several low molecular weight (LMW) agents cause occupational asthma (OA) by an IgE-independent mechanism. For example, in workers with toluene-2,4-diisocyanate (TDI)-induced OA, specific IgE is detected in only 0–40% of the workers [5–7]. This implies that, if the function of B lymphocytes would be limited to the secretion of IgE antibodies, they would not play a major role in chemical-induced OA. Yet, consistent increases in CD19^+^ B lymphocytes are observed in models of chemical-induced asthma [8–12].

Recently, De Vooght *et al*. transferred CD19^+^ B lymphocytes from TDI sensitized mice into naïve mice. Three days later, these mice exhibited AHR and a neutrophilic lung inflammation after a single TDI-challenge. Moreover, transferring CD19^+^ B lymphocytes from TDI-sensitized wild type mice into B cell KO mice or SCID (severe combined immunodeficiency) mice also resulted in AHR and neutrophilic lung inflammation after a single TDI-challenge, suggesting a potential role for B lymphocytes without the need or even presence of T lymphocytes [13]. Furthermore, B lymphocytes from TDI-sensitized mice expressed surface markers such as MHC II, and co-stimulatory molecules, confirming the results of Lindell *et al*., and implying a potential role of antigen presentation for B cells [4]. So, B lymphocytes from sensitized mice appear to carry a ‘sensitization signature’, which allows them to generate an asthma-like response after a specific allergen challenge in naïve mice who received an adoptive transfer of B lymphocytes from sensitized mice.

We compared the proteome of auricular CD19^+^ B lymphocytes from TDI-sensitized versus control mice, in order to identify a possible ‘sensitization signature’.

## Materials and Methods

All experiments were performed with approval of the KU Leuven Ethical Committee on animal experiments (project number: 066/2011).

### Reagents

Toluene-2,4-diisocyanate (TDI) (98%; Fluka, CAS 584-84-9) and acetone were obtained from Sigma-Aldrich (Bornem, Belgium). Pentobarbital (Nembutal^®^) was obtained from Sanofi Santé Animale (CEVA, Brussels, Belgium). The vehicle (acetone/olive oil, AOO) used to dissolve TDI consisted of a mixture of 2 volumes of acetone and 3 volumes of olive oil (Selection de Almazara, Carbonell, Madrid, Spain). Concentrations of TDI are given as percent (v/v) in AOO.

### Animals

Male BALB/c mice were obtained from Harlan (Horst, The Netherlands) and were kept in a conventional animal house in filter top cages with a 12h light/dark cycle and had access to lightly acidified water and food (Trouw Nutrition, Ghent, Belgium) *ad libitum*. All mice were 6 weeks old and approximately 20 g.

### Mouse model and cell isolation

On days 1 and 8, mice received dermal applications of 0.3% TDI or vehicle (AOO, control) on the dorsum of both ears (20 μl/ear). On day 15, these mice were sacrificed and auricular lymph nodes were collected and pooled.

Cell suspensions were obtained by pushing the lymph nodes through a cell strainer (100 μm) and rinsing them with 10 ml buffer (MACS BSA Stock solution diluted 1:20 with autoMACS Rinsing Solution (Miltenyi Biotec, Utrecht, The Netherlands). Following centrifugation (1000 g, 4°C, 10 min) cells were counted using a Bürker hemocytometer. CD19^+^ B-lymphocytes were isolated with CD19^+^ microbeads (Miltenyi Biotec, Utrecht, The Netherlands) according to manufacturer’s instructions.

### Sample preparation for 2D-DIGE

Isolated B-lymphocytes were homogenized in 200 μl of lysis buffer with protease inhibitor using a cone sonicatior (3 x 10 s with 30 s on ice in between). Following centrifugation (1000 g, 4°C, 10 min), supernatants of isolated CD19^+^ B lymphocytes were removed and 200μl of lysis buffer (40 mM Tris base, pH 8.8, 7 M urea, 2 M thiourea, 4% CHAPS and 1% dithiothreitol) with protease inhibitor (40 μl of ¼ tablet Complete in 500 μl MilliQ water, per ml lysis buffer) were added. Samples were sonicated and centrifuged (13000 g, 4°C, 10 min) after which supernatants were collected. To concentrate samples, ultramembrane centrifugation with Amicon Ultra10k (cut-off 10 kDa, Merck Millipore, Darmstadt, Germany) was performed according to manufacturer’s instructions.

Prior to labelling, pH of all samples was adjusted (between pH 8–9), if necessary, by adding small amounts of lysis buffer (pH = 8.35). Afterwards samples were desalted via dialysis (Mini Dialysis kit 1 kDa cut-off, GE Healthcare, Freiburg, Germany) and concentrations were measured using the Bradford method.

### 2D-DIGE analysis and protein identification

Six biological replicates of pooled CD19^+^ B lymphocytes extracted from auricular lymph nodes of TDI-sensitized mice (n = 3) were compared with six biological replicates of pooled CD19^+^ B lymphocytes of control mice (n = 5) after CyDye DIGE Fluor minimal labelling (GE Healthcare) according to manufacturer’s instructions. A two-dimensional gel electrophoresis separation was carried out as previously described [14]. Gels were scanned with the Ettan DIGE Imager according to manufacturer’s instructions and digital images were analysed with Decyder 2D Differential Analysis 7.0 software (GE Healthcare).

Principal component analysis (PCA) was performed in the Extended Data Analysis module. PCA is a mathematical procedure that converts a set of variables (here proteome maps) into a new coordinate system such that the greatest variance is depicted on the first coordinate (called the first component or PC1), the second greatest variance on the second coordinate (PC2), and so on. So, if proteome maps of B cells from TDI-sensitized versus non-sensitized mice are sufficiently different, they should be clearly separated by projection on PC1. In other words, a PC1 value equal to 80% implies that 80% of the variance present in the dataset is explained by PC1 (e.g. control vs. TDI).

Spots of interest were manually excised under a laminar flow hood from silver stained preparative gels containing 150 μg of protein. Removal of silver and trypsin digestion were performed as previously described [14]. Tryptic digests were redissolved in 50 μl of 2% acetonitrile/0.1% aqueous trifluoroacetic acid and concentrated and desalted using Millipore Ziptips_C18_ (15 μm), according to manufacturer’s instructions. Elution was done with 4.5 μl of 70% acetonitrile/0.1% formic acid. 1 μl was then spotted together with 1 μl of a saturated solution of α-cyano-4-hydroxy-cinnamic acid (matrix) in acetone on a MALDI target plate.

Peptide mass fingerprinting was performed on a matrix-assisted laser desorption ionisation tandem time-of-flight (MALDI-TOFTOF) mass spectrometer in positive ion reflectron mode (Ultraflex II, Bruker Daltonics, Bremen, Germany).

Protein database (NCBInr database or SwissProt—*Mus musculus* taxonomy) searching was performed with peptide mass fingerprinting on an ‘in-house’ Mascot server (Matrix Science, London, UK). Trypsin was selected as an enzyme, peptide tolerance was set to 0.2 Da, one missed cleavage per peptide was allowed, carbamidomethylation of cysteine, as a result of the equilibration with iodoacetamide, was set as a fixed modification and methionine oxidation was set as variable modification. The probability-based MOWSE (Molecular Weight Search) scores greater than the given cut-off value were considered as significant (p < 0.05).

The identified proteins were classified according to their biological function retrieved from Gene Ontology.

### Verification of differentially expressed proteins

Two of the identified immune-related proteins, Cyclophilin A (CypA) and Cofilin 1 (Cof1), that were included in the list of proteins contributing to the separation of the TDI-treated group and AOO-treated group in the PCA were verified via Western blotting in B lymphocytes obtained from a new, independent set of similarly treated TDI- and AOO-treated mice (n = 10/group). Briefly, proteins were loaded and separated on 4–12% Bis/Tris Midi-gels (Invitrogen, Merelbeke, Belgium) and subsequently transferred to a PVDF membrane (iBlot, Gel Transfer Stack, Invitrogen). Membranes were blocked (1–2 h, 5% blocking agent, GE Healthcare) and incubated overnight with primary antibody (LSP-1: 1/1000, goat Ab, Santa Cruz Biotechnology; CypA: 1/1000, mouse Ab, Santa Cruz Biotechnology; GAPDH, internal standard, 1/200000, mouse Ab, Dako). Following secondary Ab incubation (LSP-1: 1/50000, donkey anti-goat IgG, Santa Cruz Biotechnology; Cof1 and GAPDH: 1/100000, goat anti mouse IgG, Dako) protein bands were visualized using chemiluminescence detection (Supersignal West Dura, Thermo Scientific) on ECL hyperfilm (GE Healthcare). The protein bands were semiquantitatively evaluated by densitometry (ImageQuant TL v2009, GE Healthcare).

### Statistical analysis

Graphpad Prism 5.01 (Graphpad Software Inc) was used for statistical analysis of the Western blot data. All data were normally distributed (tested using Kolmogorov-Smirnov). An unpaired t-test was used to compare both groups (AOO vs. TDI). A level of p<0.05 was considered significant.

## Results

### 2D-DIGE analysis and protein identifications

**Fig 1:**
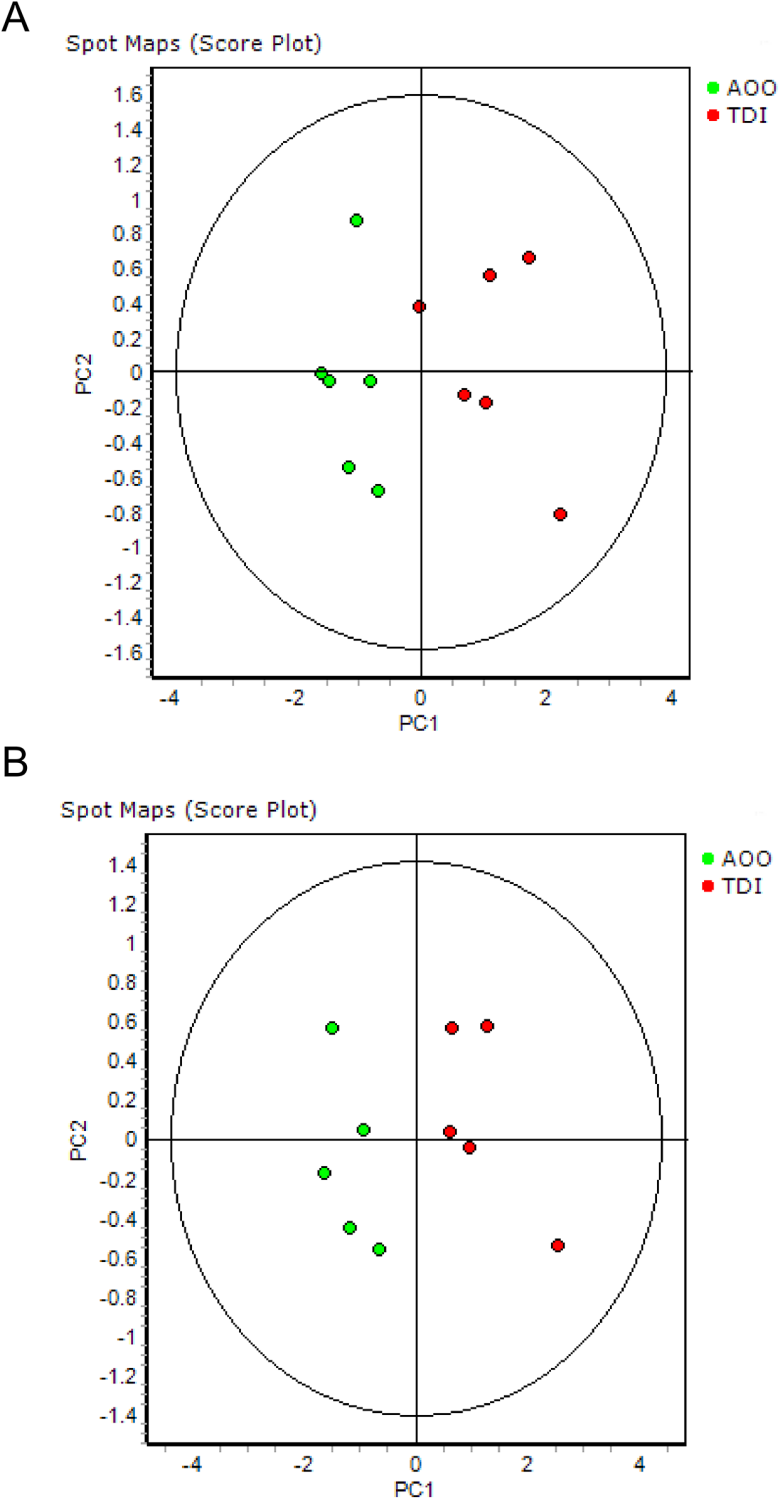
Principal component analysis (PCA). PCA of the proteome maps of sensitized and non-sensitized CD19^+^ B lymphocytes. **(A)** PCA of all six biological replicates. **(B)** PCA of five biological replicates. Green dots correspond to AOO-treated mice (controls; n = 5/group). Red dots correspond to TDI-sensitized mice (n = 3/group).

The proteomes of six CD19^+^ B lymphocyte extracts obtained from pooled auricular lymph nodes from TDI-sensitized mice were compared with the proteomes of six CD19^+^ B lymphocyte extracts obtained from AOO-treated control mice via 2D-DIGE. Proteome analysis revealed 38 differentially expressed proteins (p<0.01), of which 15 proteins were upregulated and 23 were downregulated in B lymphocytes from TDI-sensitized mice. Principal component analysis (PCA; Fig 1A) separated both the TDI and AOO proteome maps based on the differentially expressed proteins (p<0.01). Only one TDI proteome map was not clearly separated. PC1 and PC2 values were equal to 74.7% and 12.7% respectively. Removing the gel that contained the ‘aberrant’ TDI proteome map from the analysis, resulted in a complete separation of both groups, with PC1 and PC2 values equal to 84% and 8.6% respectively (Fig 1B).

**Fig 2:**
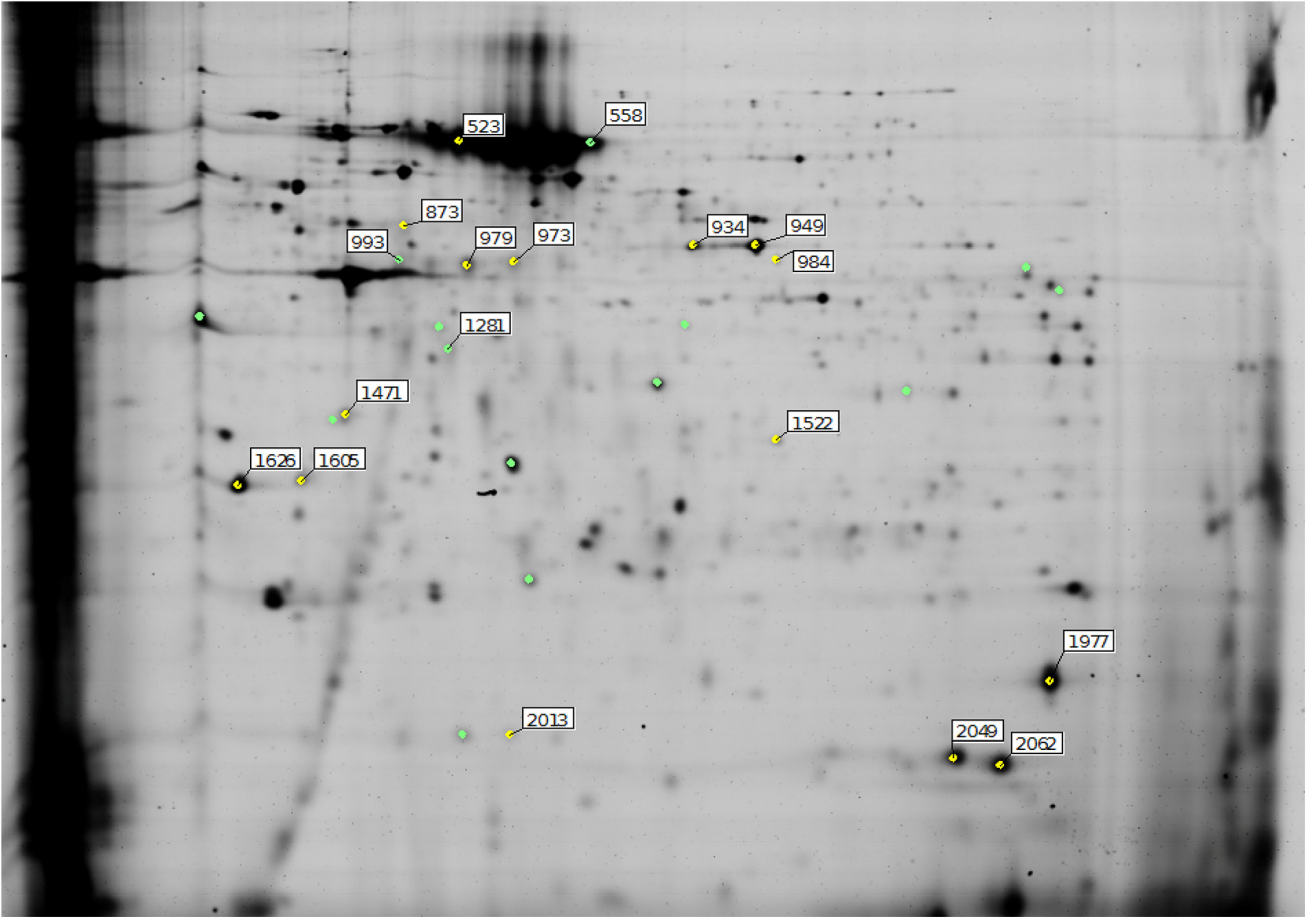
Representation of the identified differentially expressed proteins on a raw 2D-DIGE gel image.

**Fig 3:**
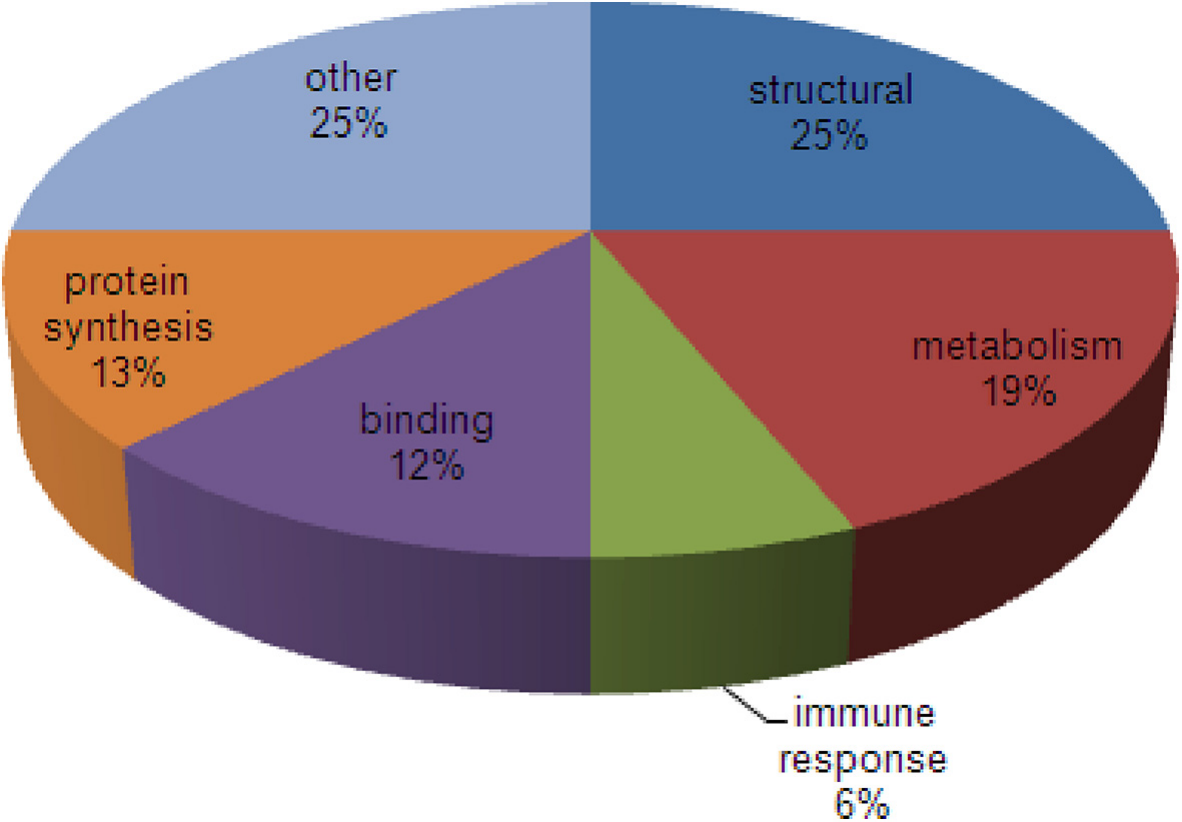
Classification of the identified differentially expressed proteins. The differentially expressed proteins in CD19^+^ B lymphocytes from sensitized versus non-sensitized mice were classified according to biological function (retrieved from Gene Ontology).

Using, peptide mass fingerprinting 16 differentially expressed proteins were identified (Table 1, Fig 2, S1–S15 Figs). The identified proteins were grouped in classes according to their biological function retrieved from Gene Ontology (www.geneontology.org, Fig 3). Proteins were classified as structural proteins (25%), proteins involved in metabolism (19%), protein synthesis (13%) or binding proteins (12%). One protein, zinc finger CCHC domain containing protein 11, is involved in immunity. The remaining proteins were involved in other biological functions (25%).

**Table 1:** Differentially expressed proteins in CD19^+^ B cells from TDI-sensitized versus non-sensitized mice (p<0.01).

*Gene Ontology Spot no*.	*Name*	*No*. *of Spots*	*Accession ID*	*Score*	*Seq Cov (%)*	*Peptides matched*	*Fold Change*	*pI* *	*Mw (Da)* *
**Structural**									
1977	cofilin 1	1	P18760	76	65	12	1.9834	8.22	18776
873	dynamin 1	1	P39053	66	18	14	-1.8080	6.57	98140
993	beta actin	1	P60710	70	38	12	-1.8503	5.23	42053
979	nebulin-related-anchoring protein (N-RAP)	1	Q80XB4	70	30	35	-2.1183	9.27	133899
**Metabolism**									
1471	alcohol dehydrogenase [NADP+]	1	Q9JII6	75	33	13	2.2563	6.90	36792
934–949	alpha-enolase	2	P17182	166	50	24	1.6560	6.37	47453
1281	dTDP-D-glucose 4,6-dehydratase	1	Q8VDR7	56	24	11	-1.8157	5.92	40857
**Immune response**									
558	zinc finger CCHC domain containing protein 11	1	B2RX14	81	26	25	-2.1068	6.72	114480
**Binding**									
973	vinculin	1	Q64727	55	26	26	-1.8792	5.77	117215
523-973-979	centromere protein C 1 (CENP-C)	3	P49452	57	29	27	-2.1183	9.71	102676
**Protein synthesis**									
2049–2062	cyclophilin A	2	P17742	96	64	15	2.8536	7.74	18131
984	elongation factor Tu	1	Q8BFR5	114	61	23	1.5235	8.59	50240
**Other**									
1626	rho GDP dissociation inhibitor (GDI) beta	1	Q61599	70	59	10	2.6210	4.97	22894
2013	proteasome 26S subunit	1	P46471	78	24	16	2.4681	5.25	40580
1522	sterile alpha motif domain-containing protein 14	1	Q8K070	67	26	10	2.1569	9.51	45075
1605	rho GDP dissociation inhibitor (GDI) alpha	1	Q99PT1	98	47	14	1.8416	5.20	22991

Proteins are ranked according ontology, followed by most upregulated to most downregulated. Individual mass spectra can be found in S1 to S15 Figs.

* pI and Mw are depicted as theoretical values.

### Western blot verification

**Fig 4:**
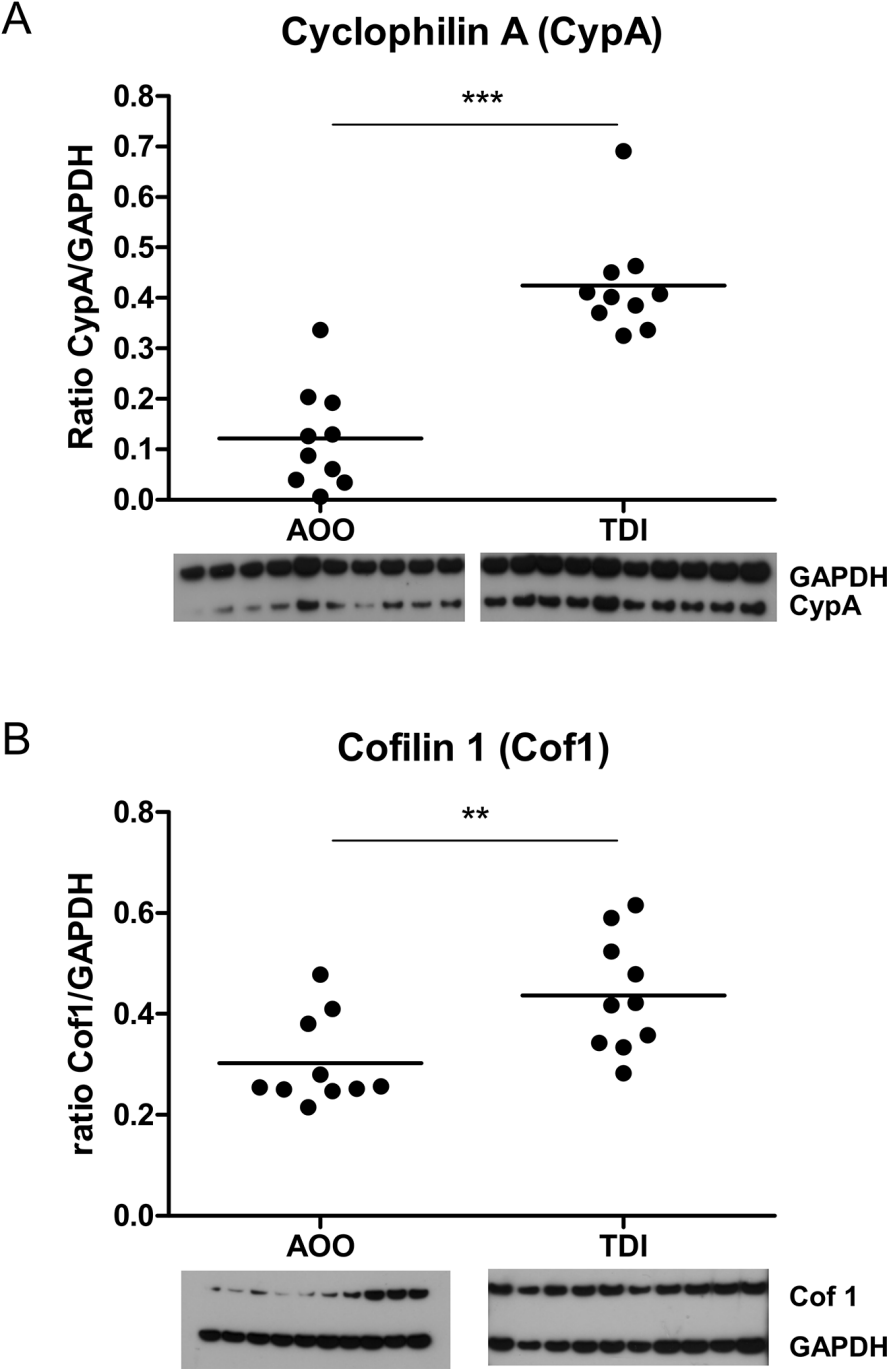
Validation of differentially expressed proteins using Western blotting. **(A)** Cyclophilin A (CypA) and **(B)** Cofilin 1 (Cof1) validation in CD19^+^ B lymphocytes using Western blotting. Validation experiments were performed in a separate set of mice. Figure shows individual mice and group means. n = 10; **p<0.01, ***p<0.001.

We verified two of the differentially expressed proteins resulting from the 2D-DIGE analysis via Western blotting in an independent group of TDI-treated and AOO-treated mice. We selected cyclophilin A (CypA) and cofilin 1 (Cof1) for verification based on two criteria: (1) biologically relevance in the process of sensitization and (2) contribution to the discrimination between TDI- and AOO-treatment in the principal component analysis (PCA). Western blotting confirmed the significant up-regulated expression of both CypA and Cof1 in B lymphocytes of TDI-sensitized mice (Fig 4A and 4B).

## Discussion

In this study, we applied a proteomics approach to compare the proteomes of isolated sensitized and non-sensitized CD19^+^ B lymphocytes in a well-established and characterized mouse model of chemical-induced asthma [9,14–19]. In previous isolation experiments, the purity of the isolated CD19^+^ B lymphocytes was verified several times via FACS. The combined impurity (T cells, dendritic cells, …) was always less than 5% and, therefore, it was not checked in this study [13]. Using 2D-DIGE, we found significant differences in the respective proteomes, with the identification of several proteins that can be linked to the onset of chemical-induced asthma.

An important role has long been reserved for T lymphocytes and the cytokines they produce in the development of allergic asthma and OA, whereas the role of B lymphocytes in allergic asthma or OA was thought to be restricted to the production of antibodies. Since, several LMW agents (e.g. diisocyanates and persulfate salts) causing OA probably do not act via specific IgE antibodies, it might at first site suggest that B lymphocytes are merely bystanders. Yet, recent studies have rejuvenated the interest in the role of B lymphocytes. In a mouse model of cockroach-induced asthma, Lindell *et al*. showed that B lymphocytes can act as antigen presenting cells (APC), presenting antigen to naïve T lymphocytes [20]. Our research group also confirmed an increased MHCII expression on the surface of B lymphocytes of TDI-sensitized mice [13]. Moreover, allergen-induced AHR was significantly attenuated in B cell KO mice compared to control mice [4]. In our mouse model of chemical-induced asthma, similar effects were seen. Naïve wild type, B cell KO or SCID mice, that received CD19^+^ B lymphocytes from TDI-sensitized mice were challenged three days later with TDI, resulting in AHR and a neutrophilic lung inflammation [13]. The observed time course (three days) points against a process depending on IgE production by mature plasma cells. Moreover, the glycoprotein CD19 is only expressed on the surface of early pre-B lymphocytes and not on IgE producing plasma cells; consequently no immunoglobulin producing cells were transferred, thus again supporting antibody-independent functions for B lymphocytes [21]. So far, it remains unknown which features differentiate sensitized from non-sensitized B lymphocytes.

Our 2D-DIGE study shows significant differences in the proteome of B lymphocytes from TDI-sensitized mice compared with non-sensitized mice. In fact PCA can discriminate TDI-sensitized from non-sensitized mice based on their proteome maps. PCA highlighted one aberrant proteome map among the TDI-group (Fig 1), most likely resulting from a technical issue (e.g. imperfect CyDye labeling) and not as a result of treatment, as lymphocytes of auricular lymph nodes from three animals were pooled. Excluding the gel containing this proteome map from the PCA analysis resulted in an even stronger discrimination of the two groups (PC1 = 84%, PC2 = 8.6%).

We found a nearly three-fold upregulation of cyclophilin A (CypA) in B lymphocytes of TDI-sensitized mice, which we confirmed in an independent Western Blot experiment. CypA is a member of the cyclophilins, a family of intracellular proteins found in all organisms and in all human tissues. In humans, they are mainly secreted in response to inflammatory stimuli such as LPS or reactive oxygen radicals [22,23]. CypA is a potent chemoattractant for monocytes, neutrophils, eosinophils and T lymphocytes. High levels of CypA were found in the lungs of mice with ongoing pulmonary neutrophilia induced by LPS [24]. In rheumatoid arthritis patients, a direct correlation was described between levels of CypA and the number of neutrophils in the synovial fluid [25]. Stemmy *et al*. showed that blocking the function of CypA in chronic allergic asthma decreased the number of persisting leukocytes drastically, accompanied by a significant reduction in AHR to methacholine [26]. Recently, we also showed that transfer of B lymphocytes from sensitized mice into naïve mice can lead to a neutrophilic lung inflammation after challenge [13]. We speculate on a possible role for CypA in the recruitment of neutrophils to the lungs of TDI-treated mice. Macrophage inflammatory protein 2 (MIP-2), the mouse analogue of the human pro-inflammatory cytokine IL-8, is a potent neutrophil chemoattractant. Together with CypA, MIP-2 was demonstrated to have a synergistic effect on neutrophil migration [27]. In humans, CypA induces the secretion of the pro-inflammatory cytokine IL-8. In mice, augmented levels of MIP-2 are found in the BAL fluid of TDI-asthmatic mice, suggesting a similar effect [9].

Cofilin 1 (Cof1), nearly 2-fold upregulated in B lymphocytes from TDI-sensitized mice, belongs to a family of actin-binding proteins and is ubiquitously expressed in all eukaryotic cell types. Cof1 is able to promote rearrangements in the actin cytoskeleton. T lymphocytes need a dynamic cytoskeleton to exert their function in the adaptive immune system, namely patrolling the body in the search for pathogen-derived peptides presented by antigen presenting cells [28,29]. To this end, the round shape of a T lymphocyte converts towards a more flattened shape, enabling T lymphocytes to crawl over APCs screening for antigens. Once a specific antigen is recognized, T lymphocytes further adjust their morphology, building up a close contact zone to finally become activated [29]. Here we found an upregulation of Cof1 in CD19^+^ B lymphocytes of TDI-sensitized mice. Recently, the antigen presenting functions for B lymphocytes have been rediscovered [4]. The upregulation of Cof1 is an indirect indication of the possible antigen presenting capacities of B lymphocytes, for which dynamic actin cytoskeleton rearrangements are required.

Zinc finger CCHC domain containing protein 11 (ZCCHC11) was two-fold down regulated in TDI-sensitized B lymphocytes. Jones *et al* showed that ZCCHC11 fine tunes IL-6 production by uridylating miRNA’s [30], while Minoda *et al*, proposed that ZCCHC11 could be a unique TLR signal regulator, able to suppress activation of NF-κB [31]. The activation of the NF-κB pathway is believed to be sufficient to induce B lymphocyte-induced maturation protein-1 (Blimp-1 expression), a transcription factor crucial for the development of immunoglobulin-secreting cells. Although the role of IgE in chemical-induced asthma remains a lingering question, in previous work in mice, we found significant increases in total serum IgE levels, of which we here possibly demonstrate an early signal at the protein level, i.e. preparing CD19^+^ B lymphocytes to switch to plasma cells [9,18,32].

In conclusion, we have demonstrated significant proteomic alterations in CD19^+^ B lymphocytes from TDI-sensitized mice compared to control mice. Differences in the expression of CypA, Cof1 and ZCCHC11 can be directly correlated to functions of B lymphocytes in the process of sensitization and a subsequent asthmatic response. Further studies to functionally validate these results are encouraged.

## Supporting Information

S1 Fig protein 558: mass spectrum of protein zinc finger CCHC domain containing protein 11. (TIF)

S2 Fig protein 873: mass spectrum of protein dynamin 1. (TIF)

S3 Fig protein 949: mass spectrum of protein alpha-enolase. (TIF)

S4 Fig protein 973: mass spectrum of protein vinculin. (TIF)

S5 Fig protein 979a: mass spectrum of protein nebulin-related-anchoring protein (N-RAP). (TIF)

S6 Fig protein 984: mass spectrum of protein elongation factor Tu. (TIF)

S7 Fig protein 993: mass spectrum of protein beta actin. (TIF)

S8 Fig protein 1281: mass spectrum of protein dTDP-D-glucose 4,6-dehydratase. (TIF)

S9 Fig protein 1471: mass spectrum of protein alcohol dehydrogenase [NADP+]. (TIF)

S10 Fig protein 1522: mass spectrum of protein sterile alpha motif domain-containing protein 14. (TIF)

S11 Fig protein 1605: mass spectrum of protein rho GDP dissociation inhibitor (GDI) alpha. (TIF)

S12 Fig protein 1626: mass spectrum of protein rho GDP dissociation inhibitor (GDI) beta. (TIF)

S13 Fig protein 1977: mass spectrum of protein cofilin 1. (TIF)

S14 Fig protein 2013: mass spectrum of protein proteasome 26S subunit. (TIF)

S15 Fig protein 2049: mass spectrum of protein cyclophilin A. (TIF)
