# Supplementary figures and images for: Proteomic Alterations in B Lymphocytes of Sensitized Mice in a Model of Chemical-Induced Asthma
Source: PLoS One. 2015 Sep 23;10(9):e0138791. doi: 10.1371/journal.pone.0138791 (PMC4580316; doi:10.1371/journal.pone.0138791)

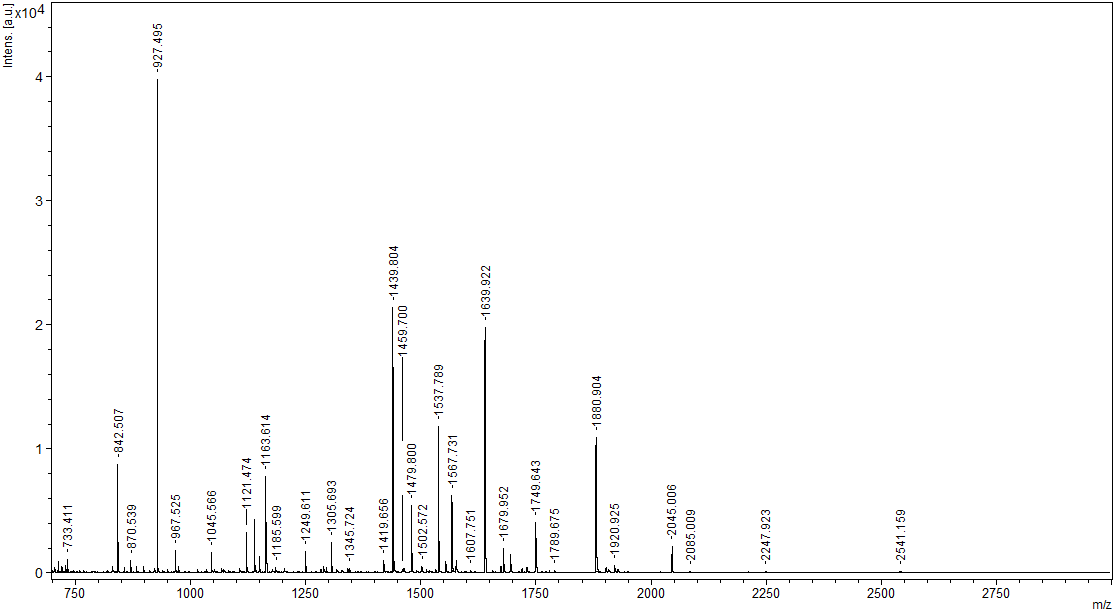

Supplement: S1 Fig — (TIF) [file pone.0138791.s001.tif]

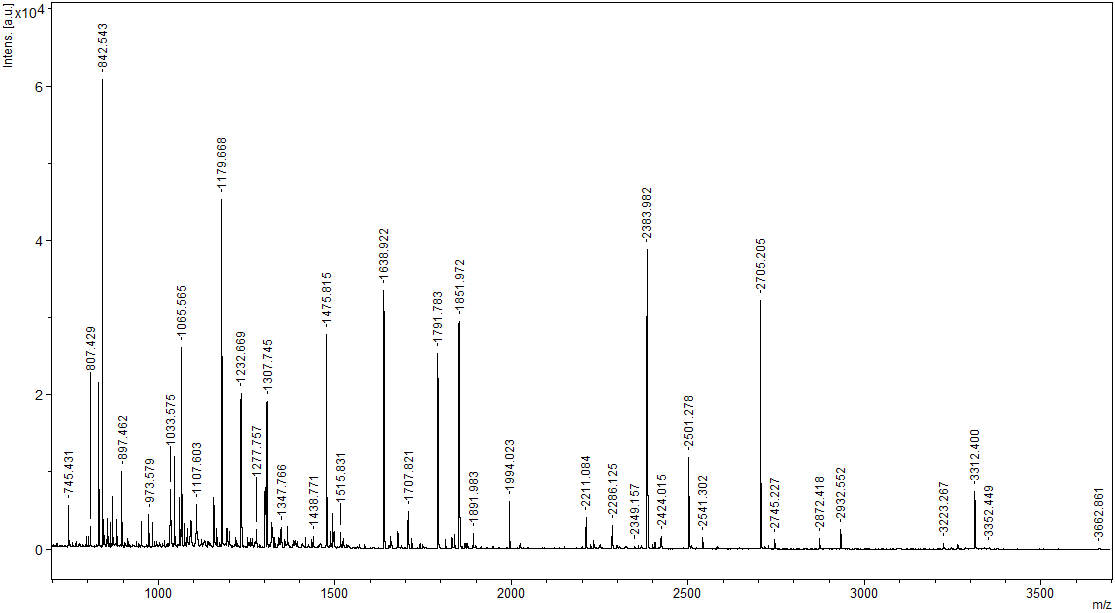

Supplement: S2 Fig — (TIF) [file pone.0138791.s002.tif]

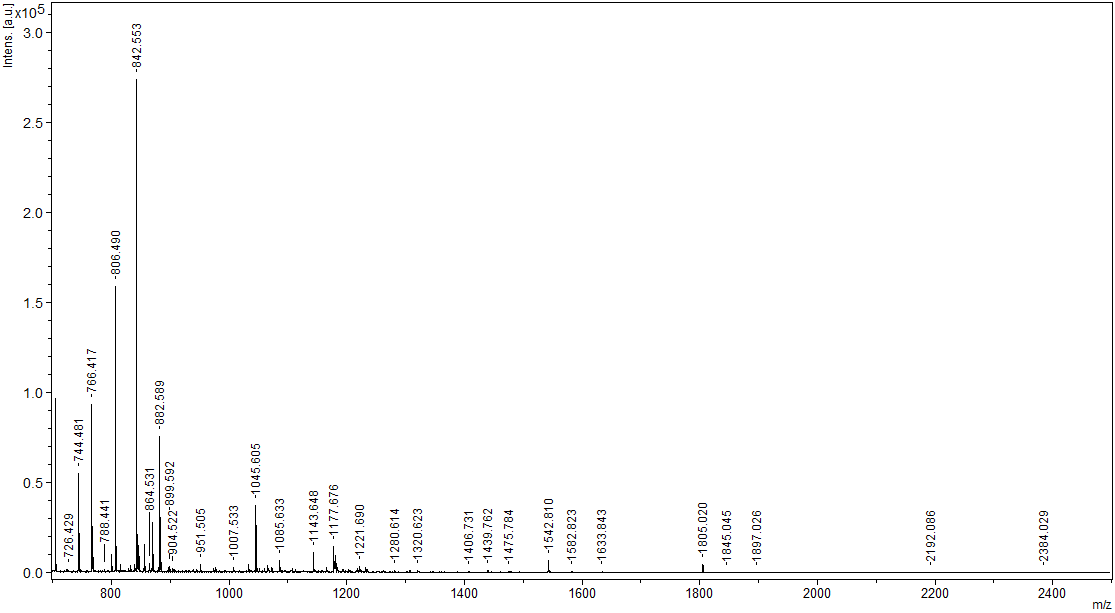

Supplement: S3 Fig — (TIF) [file pone.0138791.s003.tif]

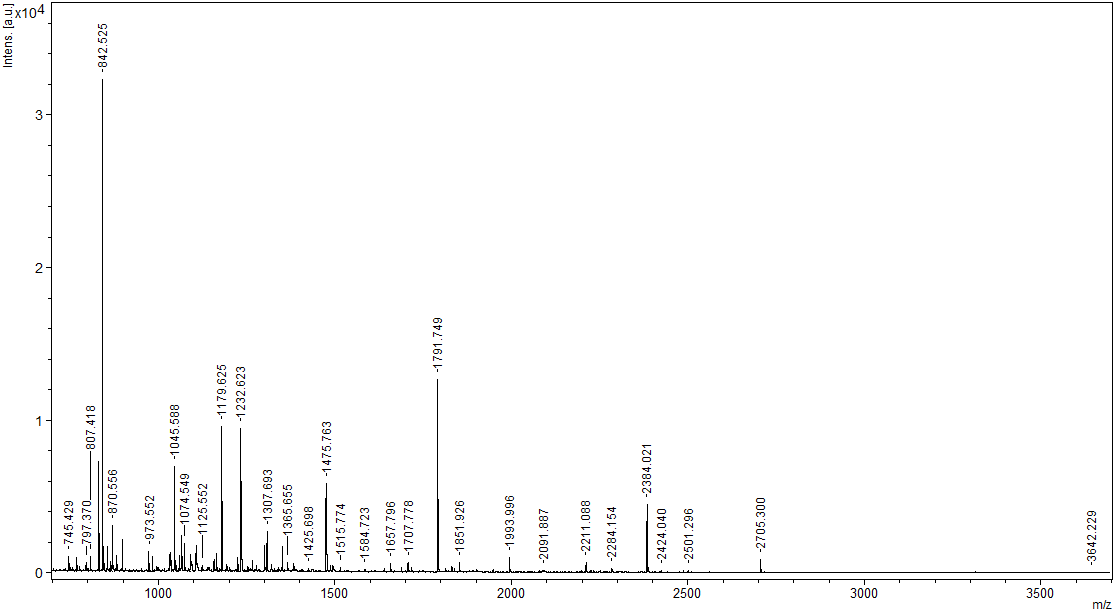

Supplement: S4 Fig — (TIF) [file pone.0138791.s004.tif]

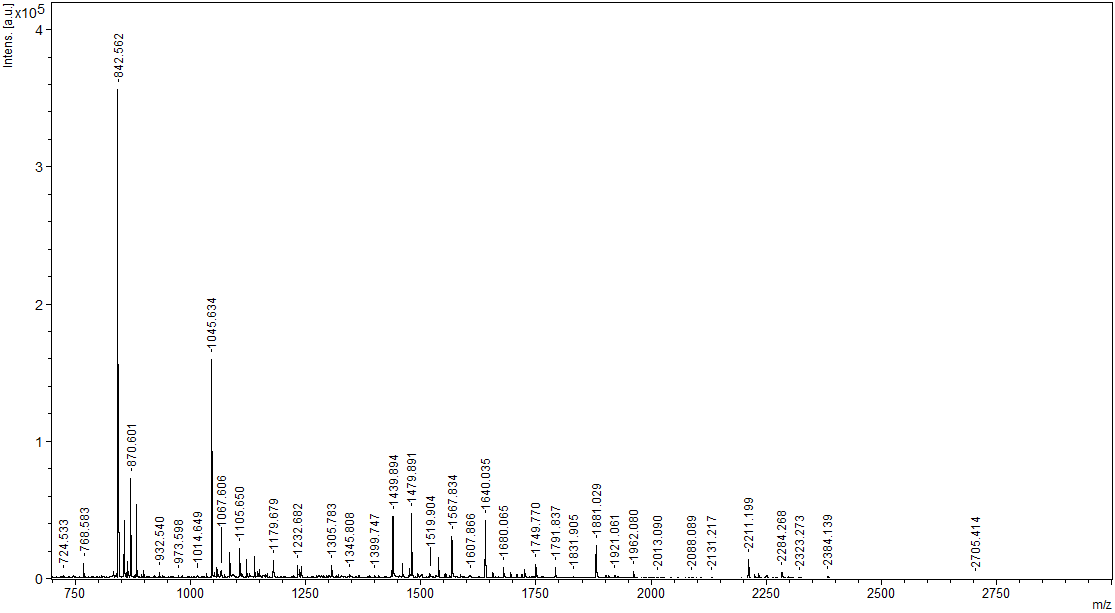

Supplement: S5 Fig — (TIF) [file pone.0138791.s005.tif]

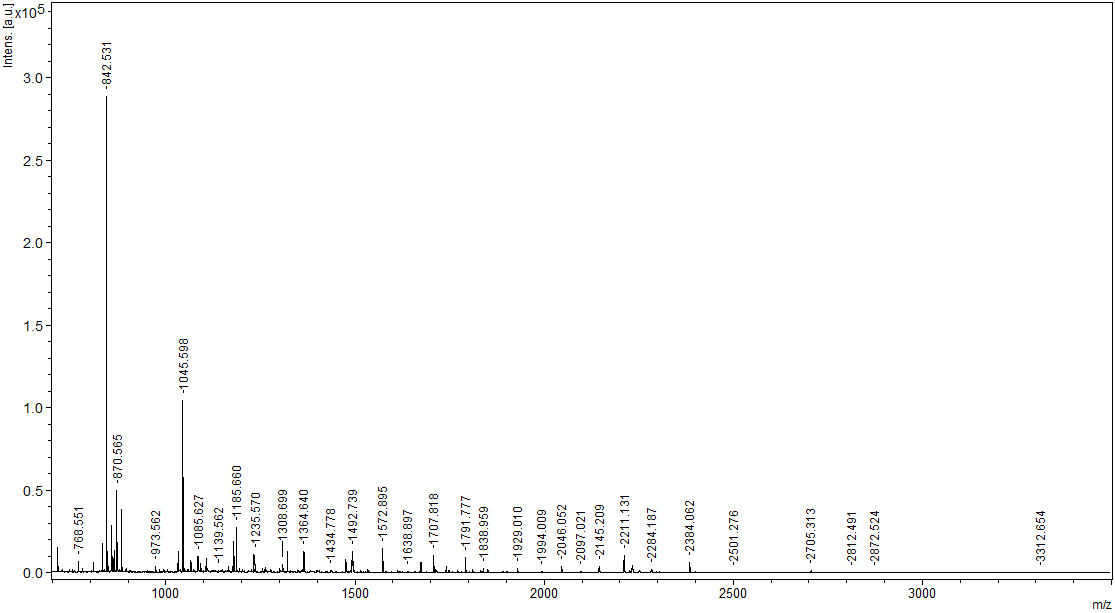

Supplement: S6 Fig — (TIF) [file pone.0138791.s006.tif]

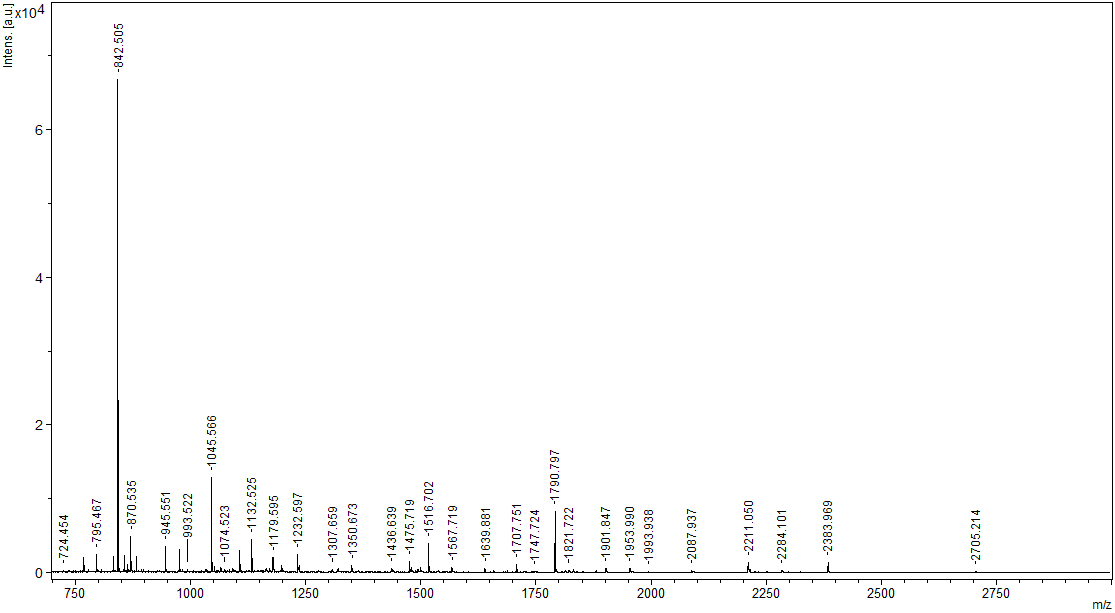

Supplement: S7 Fig — (TIF) [file pone.0138791.s007.tif]

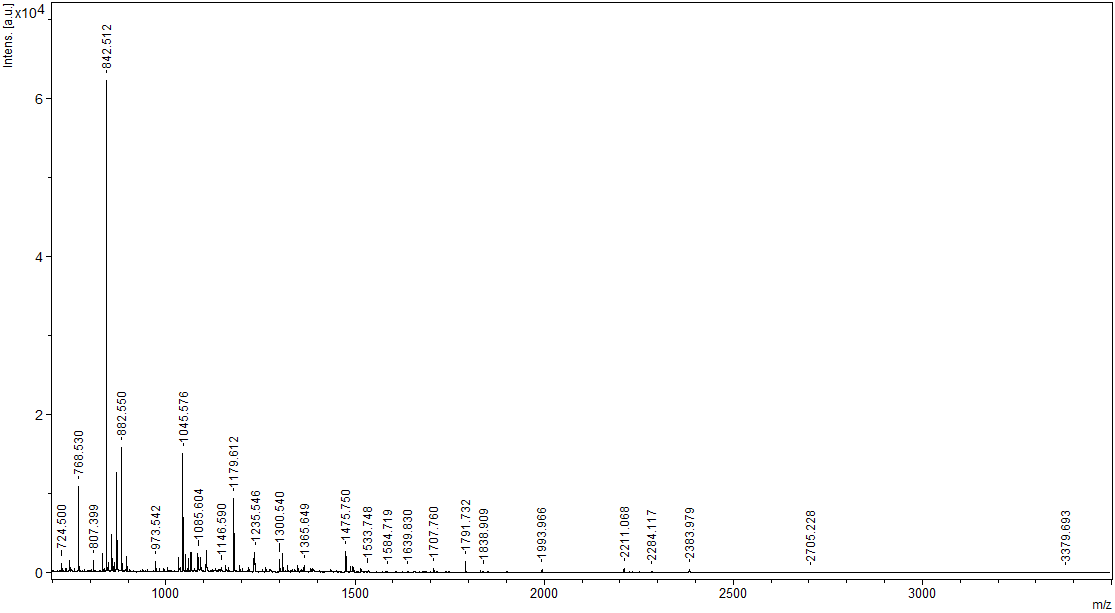

Supplement: S8 Fig — (TIF) [file pone.0138791.s008.tif]

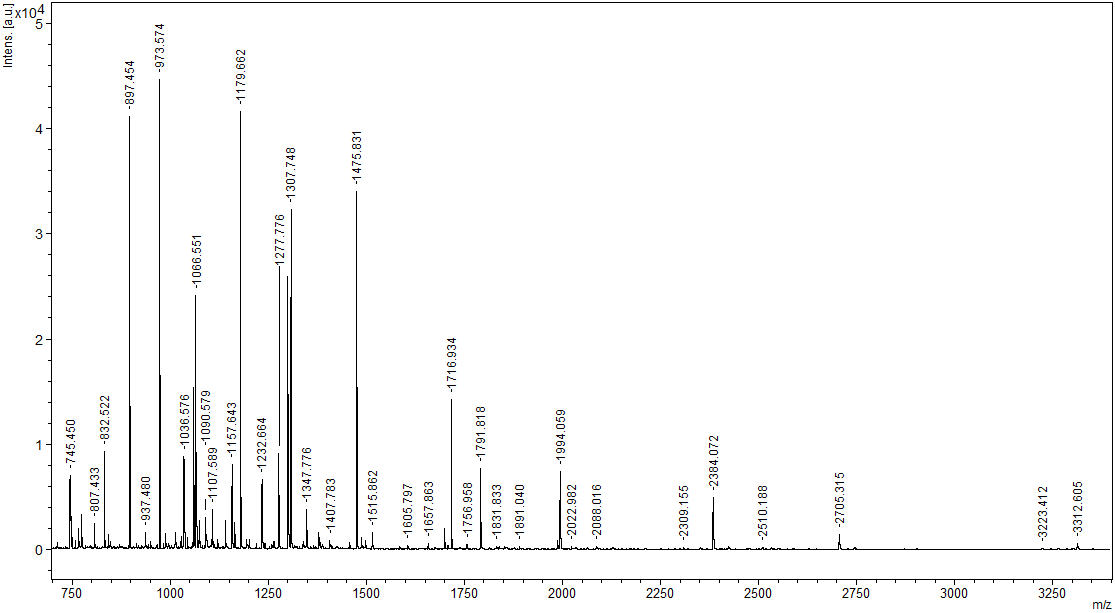

Supplement: S9 Fig — (TIF) [file pone.0138791.s009.tif]

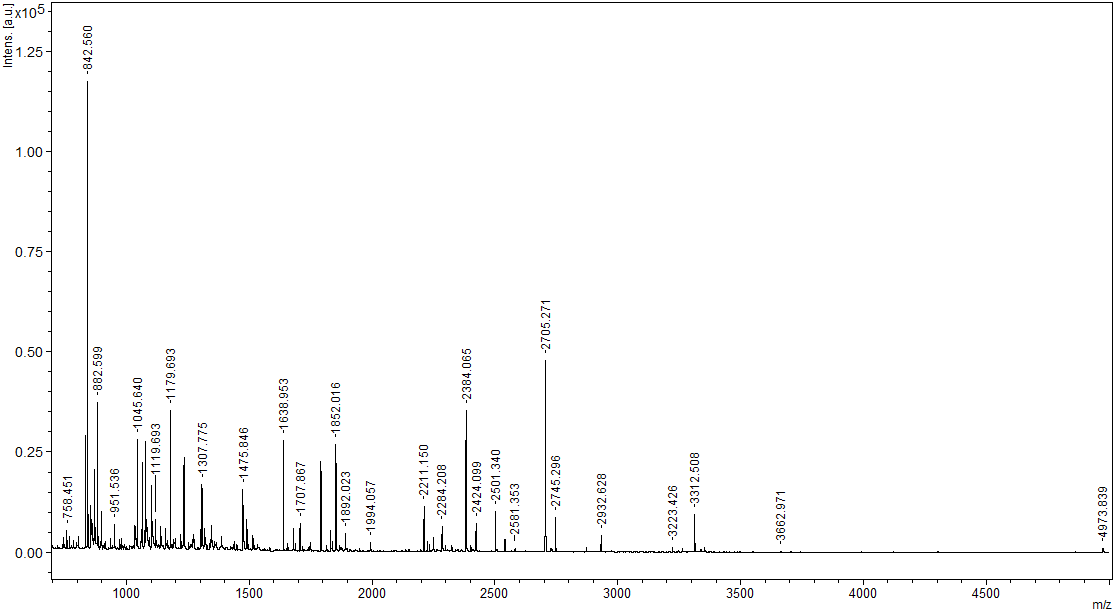

Supplement: S10 Fig — (TIF) [file pone.0138791.s010.tif]

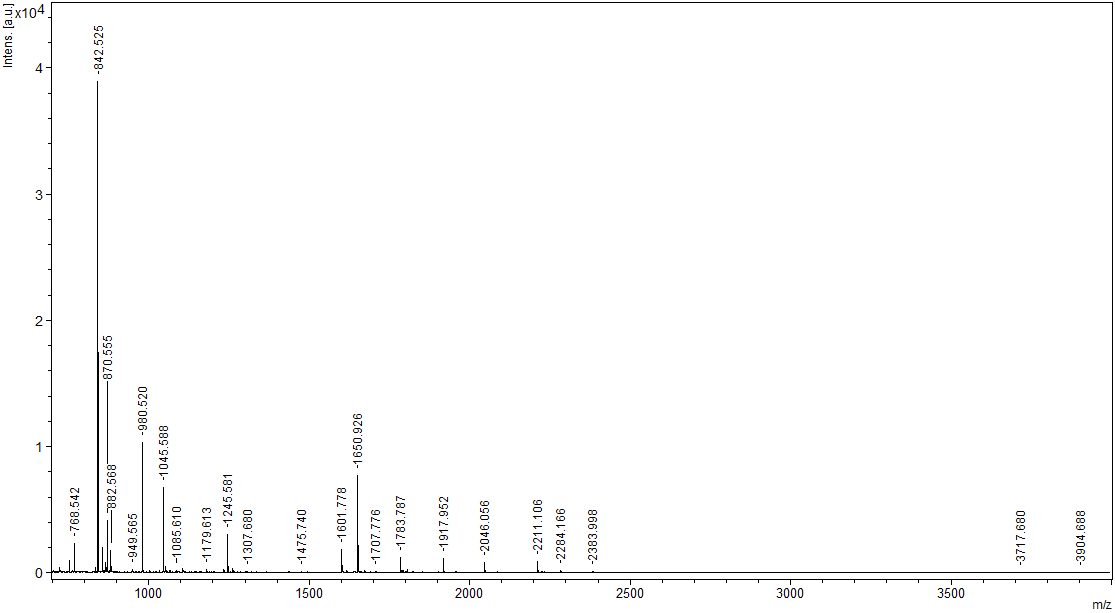

Supplement: S11 Fig — (TIF) [file pone.0138791.s011.tif]

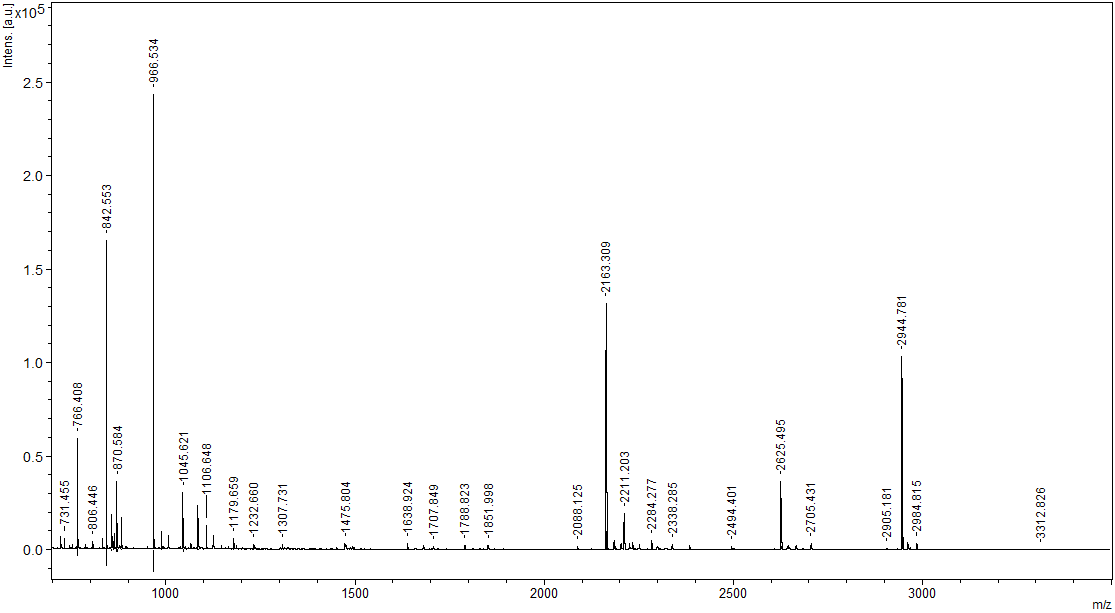

Supplement: S12 Fig — (TIF) [file pone.0138791.s012.tif]

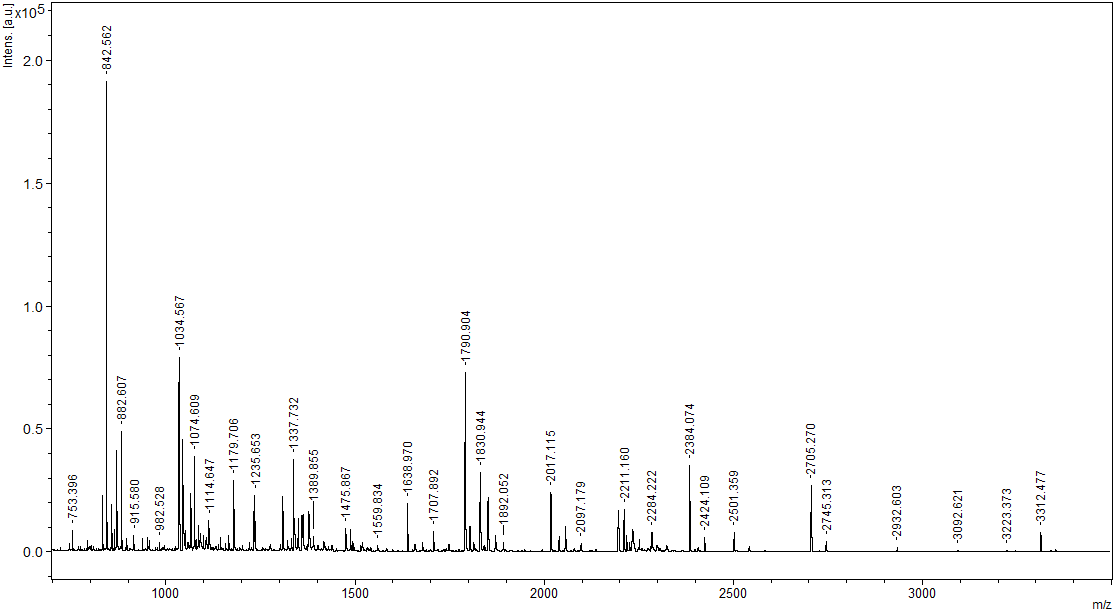

Supplement: S13 Fig — (TIF) [file pone.0138791.s013.tif]

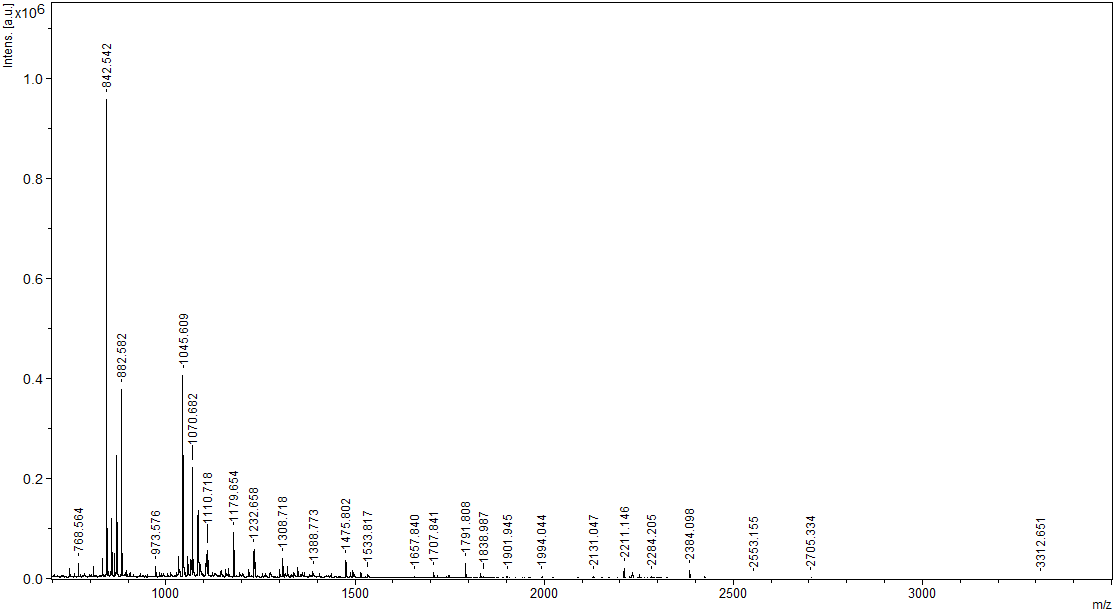

Supplement: S14 Fig — (TIF) [file pone.0138791.s014.tif]

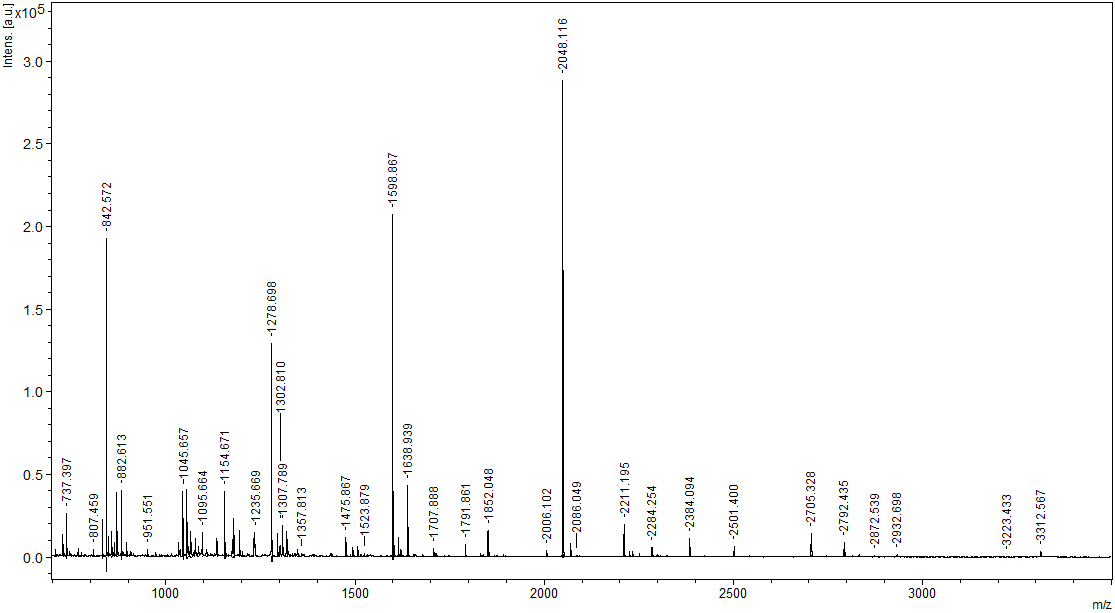

Supplement: S15 Fig — (TIF) [file pone.0138791.s015.tif]
